# Low Oxygen Concentration Reduces *Neisseria gonorrhoeae* Susceptibility to Resazurin

**DOI:** 10.3390/antibiotics13050395

**Published:** 2024-04-26

**Authors:** Justin Rice, Jordan Gibson, Emily Young, Kendall Souder, Kailee Cunningham, Deanna M. Schmitt

**Affiliations:** Department of Biomedical Sciences, West Liberty University, West Liberty, WV 26074, USA

**Keywords:** oxygen concentration, hypoxia, antibiotic susceptibility, resazurin, *Neisseria gonorrhoeae*, gonorrhea, minimal inhibitory concentration

## Abstract

*Neisseria gonorrhoeae* has developed resistance to every antibiotic currently approved for the treatment of gonorrhea, prompting the development of new therapies. The phenoxazine dye resazurin exhibits robust antimicrobial activity against *N. gonorrhoeae* in vitro but fails to limit vaginal colonization by *N. gonorrhoeae* in a mouse model. The lack of in vivo efficacy may be due to oxygen limitation as in vitro susceptibility assays with resazurin are conducted under atmospheric oxygen while a microaerophilic environment is present in the vagina. Here, we utilized broth microdilution assays to determine the susceptibility of *N. gonorrhoeae* to resazurin under low and atmospheric oxygen conditions. The minimal inhibitory concentration of resazurin for multiple *N. gonorrhoeae* clinical isolates was significantly higher under low oxygen. This effect was specific to resazurin as *N. gonorrhoeae* was equally susceptible to other antibiotics under low and atmospheric oxygen conditions. The reduced susceptibility of *N. gonorrhoeae* to resazurin under low oxygen was largely attributed to reduced oxidative stress, as the addition of antioxidants under atmospheric oxygen mimicked the reduced susceptibility to resazurin observed under low oxygen. Together, these data suggest oxygen concentration is an important factor to consider when evaluating the efficacy of new antibiotics against *N. gonorrhoeae* in vitro.

## 1. Introduction

Gonorrhea is the second most common sexually transmitted bacterial infection with over 80 million new cases reported annually [1]. Many individuals infected with *Neisseria gonorrhoeae* remain asymptomatic, and these undiagnosed and/or untreated gonococcal infections can cause severe reproductive complications, particularly for women, including pelvic inflammatory disease, ectopic pregnancy, and infertility [2,3,4]. Furthermore, the management and treatment of gonorrhea has been complicated by the increased prevalence of multidrug resistant *N. gonorrhoeae* strains.

Antimicrobial resistance in *N. gonorrhoeae* first emerged in the 1940s against sulphonamides; and has continued to expand over the past 80 years, affecting antibiotics such as beta-lactams, tetracyclines, aminoglycosides, macrolides, and more recently, quinolones [5,6,7]. Currently, extended-spectrum cephalosporins (ceftriaxone and cefixime) are the only first-line empirical monotherapy recommended for treatment of uncomplicated gonorrhea in most countries [8,9]. However, over the past decade, there have been increasing reports of ceftriaxone- and cefixime-resistant *N. gonorrhoeae* strains [10,11,12]. A retrospective observational study conducted in 2017–2018 by the WHO on antibiotic resistant gonococcal isolates revealed either complete resistance or decreased susceptibility to ceftriaxone in 21 (31%) of 68 reporting countries and to cefixime in 24 (47%) of 51 reporting countries [13]. Therefore, new antibiotics must be developed to combat this rise in multidrug resistant *N. gonorrhoeae* strains.

We previously showed the phenoxazine dye resazurin has antimicrobial activity against *Francisella tularensis* and *N. gonorrhoeae* [14,15,16]. Resazurin is produced from resorcinol (1,3-dihydroxybenzene), which is obtained by the distillation of Brazilwood extract. Resazurin inhibits the growth of a broad range of *N. gonorrhoeae* strains, including multidrug-resistant clinical isolates [14]. Furthermore, resazurin significantly reduces the number of intracellular gonococcal bacteria within human endometrial cells in vitro [14]. While resazurin exhibits potent in vitro antimicrobial activity, in vivo resazurin does not limit the colonization of mice with *N. gonorrhoeae* following vaginal infection [14]. One reason for the ineffectiveness of resazurin in vivo is its interaction with plasma proteins, specifically serum albumin. Culturing *N. gonorrhoeae* in the presence of serum albumin completely diminishes the antimicrobial activity of resazurin in vitro [14]. When the structure of resazurin is modified to maintain its antimicrobial activity in the presence of serum albumin in vitro, in vivo efficacy is improved. This resazurin analog, resorufin pentyl ether, significantly decreased vaginal colonization by *N. gonorrhoeae* over time compared with mice treated with the vehicle control [14]. However, unlike the mice administered a single dose of ceftriaxone, mice treated with resorufin pentyl ether failed to clear the infection [14]. These data suggest that better modeling of the host environment in vitro would allow for more accurate assessment of lead compounds likely to have robust activity in vivo, thus reducing the number of mice used in preclinical testing. Therefore, we aimed to identify additional host factors that affect the activity of resazurin. 

Oxygen concentration has been shown to influence bacterial susceptibility to antibiotics [17,18,19,20]. Standard in vitro antimicrobial susceptibility assays are conducted under atmospheric conditions where oxygen levels are typically around 20%. However, the oxygen concentration of most mammalian tissues is around 2–9% [17,18]. Previous studies have shown that under oxygen limiting conditions, bacteria exhibit increased resistance to antibiotics. *Staphylococcus aureus* and *Klebsiella pneumoniae* demonstrated decreased sensitivity to several aminoglycosides when cultured under anoxic (0% oxygen) and hypoxic (7–9% oxygen) conditions compared with atmospheric conditions (~20% oxygen) [19]. Additionally, depending on the antibiotic, a one to four log increase in survival of wild-type MG1655 *Escherichia coli* was observed following treatment with ampicillin, gentamicin, and norfloxacin under anaerobic conditions [20]. Under hypoxic conditions (1% oxygen), the minimal inhibitory concentrations of the routinely used antipseudomonal antibiotics ceftazidime and piperacillin-tazobactam increased against *Pseudomonas aeruginosa* [21]. Furthermore, anaerobic conditions reduced the efficacy of ciprofloxacin and tobramycin against *P. aeruginosa* biofilms [22]. Considering resazurin is a redox-sensitive dye, its biological activity is likely affected by changes in oxygen concentration. Therefore, in this study, we evaluated the effects of oxygen concentration on *N. gonorrhoeae* susceptibility to resazurin. 

## 2. Results

### 2.1. Reduced Susceptibility of N. gonorrhoeae to Resazurin at Low Oxygen

**Figure 1:**
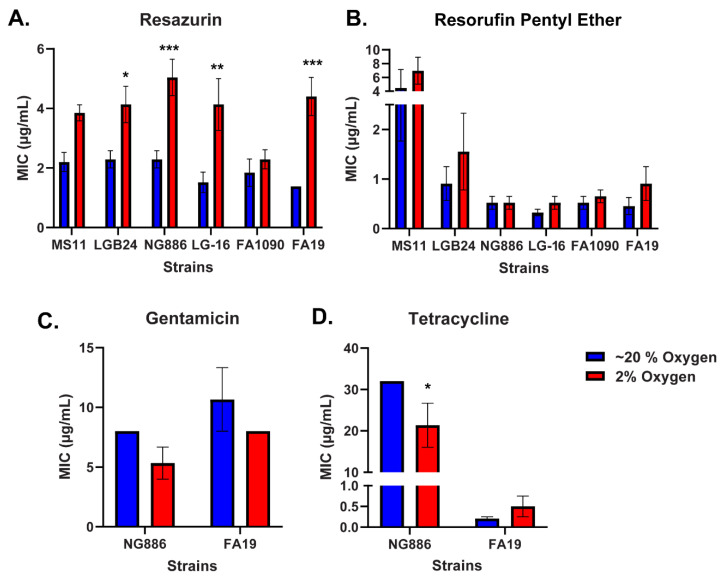
*N. gonorrhoeae* is less susceptible to resazurin, but not other antibiotics, at low oxygen. The minimal inhibitory concentrations (MICs) of resazurin (**A**), resorufin pentyl ether (**B**), tetracycline (**C**), and gentamicin (**D**) for select *N. gonorrhoeae* clinical isolates were determined under ~20% and 2% oxygen by a modified broth microdilution assay. The MIC reported for each strain is the lowest concentration of each compound that prevents visible growth. Data shown are mean ± SEM from at least three (**B**–**D**) or five (**A**) independent experiments. No error bars are present if the SEM is equal to zero. Statistically significant differences in MIC are determined by two-way ANOVA followed by Tukey’s multiple comparisons test for resazurin and RPE (**A**,**B**) and Sidak’s multiple comparison test for gentamicin and tetracycline (**C**,**D**) (*, *p* < 0.05; **, *p* < 0.01; ***, *p* < 0.001 comparing 2% to ~20% oxygen for each strain).

Resazurin exhibits robust antimicrobial activity in vitro but poor efficacy in vivo [14]. To examine the role oxygen concentration may play on the antibiotic efficacy of resazurin, the susceptibility of six different *N. gonorrhoeae* clinical isolates to resazurin was determined under atmospheric (~20%) and low (2%) oxygen conditions. Increased MICs were observed for five of the *N. gonorrhoeae* strains tested at low oxygen compared with atmospheric conditions, with FA1090 being the only strain exhibiting no change in MIC under the different oxygen conditions (Figure 1A). Next, we assessed the susceptibility of *N. gonorrhoeae* to a resazurin analog, resorufin pentyl ether (RPE), under a reduced oxygen concentration. RPE significantly reduced vaginal colonization by *N. gonorrhoeae* in a mouse model but did not completely clear the infection [14]. In contrast with resazurin, no statistically significant differences in RPE MICs were observed under 2% compared with ~20% oxygen conditions (Figure 1B). Moreover, we wanted to test whether the reduced susceptibility of *N. gonorrhoeae* to resazurin at low oxygen was observed with other clinically relevant antibiotics. As NG886 and FA19 showed the greatest differences in resazurin susceptibility at the different oxygen concentrations, we determined the gentamicin and tetracycline MICs for these strains under atmospheric (~20%) and low (2%) oxygen conditions. Gentamicin MICs for NG886 and FA19 decreased under low oxygen; however, these differences were not statistically significant (Figure 1C,D). The tetracycline MIC for NG886, but not FA19, significantly decreased between atmospheric and low oxygen levels; however, the MIC under low oxygen decreased while an increase in MIC was observed with resazurin (Figure 1D). Together, these data suggest that oxygen levels directly influence resazurin susceptibility, with *N. gonorrhoeae* being more resistant to resazurin under low oxygen conditions. 

### 2.2. MtrCDE Efflux Pump Contributes to the Reduced Susceptibility of N. gonorrhoeae to Resazurin at Low Oxygen

**Figure 2:**
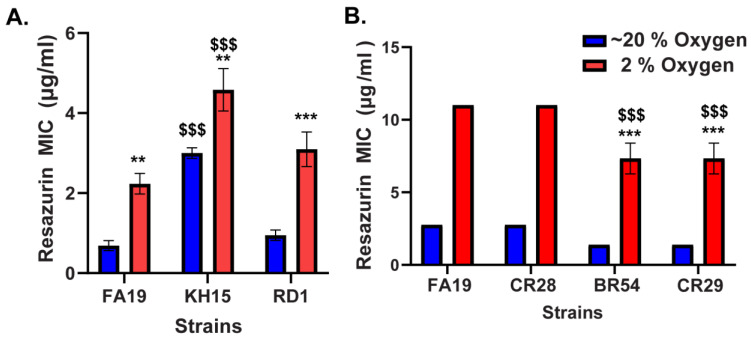
Partial contribution of the MtrCDE efflux pump in the reduced susceptibility of *N. gonorrhoeae* to resazurin at low oxygen. Resazurin MICs for select MtrCDE (**A**) and NorM *N. gonorrhoeae* mutants (**B**) are determined under ~20% and 2% oxygen by a modified broth microdilution assay. The MIC reported for each strain is the lowest concentration of each compound that prevents visible growth. Data shown are mean ± SEM from at least three independent experiments. No error bars are present if the SEM is equal to zero. Statistically significant differences in MIC are determined by two-way ANOVA followed by Tukey’s multiple comparisons test (**, *p* < 0.01; ***, *p* < 0.001 comparing 2% to ~20% oxygen for each strain; $$$, *p* < 0.001 comparing mutant to parent strain FA19 at the same oxygen concentration).

Increased antimicrobial resistance under low oxygen conditions has been noted for several bacterial strains and is associated with increased efflux pump activity [21,23,24]. For example, *Pseudomonas aeruginosa* is less susceptible to a variety of antibiotics under hypoxic conditions due to the enhanced expression of RND (Resistance-Nodulation-Division) efflux pumps [21]. Additionally, increased efflux activity of the RND type pump MdtEF resulted in enhanced drug tolerance in *E. coli* cultured under anaerobic conditions [25]. *N. gonorrhoeae* possesses five different efflux pumps: MtrCDE, FarAB-MtrE, MacAB-MtrE, NorM, and MtrF [26]. The RND type pump MtrCDE has a wide substrate range and is the most clinically important efflux system in *N. gonorrhoeae*, mediating resistance to multiple antibiotics [7,27,28]. To investigate whether the increased resistance of *N. gonorrhoeae* to resazurin under low oxygen was due to a change in efflux pump activity, we tested the resazurin susceptibility of select *N. gonorrhoeae* MtrCDE mutants. When comparing the susceptibility of the MtrCDE overexpressing strain KH15 and the MtrCDE-deficient strain RD1 to resazurin in ~20% and 2% oxygen, MICs were significantly greater under low oxygen conditions compared with atmospheric (Figure 2A). However, significantly higher resazurin MICs were observed for MtrCDE overexpressing strain KH15 in both ~20% and 2% oxygen conditions compared with the parent strain FA19 (Figure 2A). This suggests that MtrCDE may play a role in *N. gonorrhoeae* resistance to resazurin. Another *N. gonorrhoeae* efflux pump of interest is NorM, which has been shown to transport ethidium bromide and acriflavine [27,29], heterocyclic compounds that are structurally similar to resazurin. To address the contribution of NorM to the increased resistance of *N. gonorrhoeae* to resazurin in 2% oxygen, we tested the susceptibility of the NorM-deficient mutant CR28. The resazurin MIC for CR28 in 2% oxygen was 4-fold higher than the MIC in 20% oxygen with a similar difference seen in parent strain FA19 (Figure 2B). As NorM and MtrCDE share many of the same substrates [27,29], we also wanted to investigate the joint contribution of NorM and MtrCDE to the reduced susceptibility of *N. gonorrhoeae* to resazurin in low oxygen using the NorM MtrD double mutant CR29. This strain was generated from the MtrD mutant BR54. As observed with the other *N. gonorrhoeae* efflux mutants included in this study, both CR29 and BR54 had a significantly higher resazurin MIC at 2% oxygen compared with ~20% oxygen (Figure 2B). However, there was a significant decrease in the resazurin MIC for BR54 and CR29 at low oxygen compared with the parent strain FA19 (Figure 2B). Together, these data suggest the efflux pump MtrCDE contributes to, but is not solely responsible for, the enhanced resistance of *N. gonorrhoeae* to resazurin under low oxygen conditions.

### 2.3. Oxidative Stress Plays a Role in the Enhanced Susceptibility of N. gonorrhoeae to Resazurin at Atmospheric Oxygen

**Figure 3:**
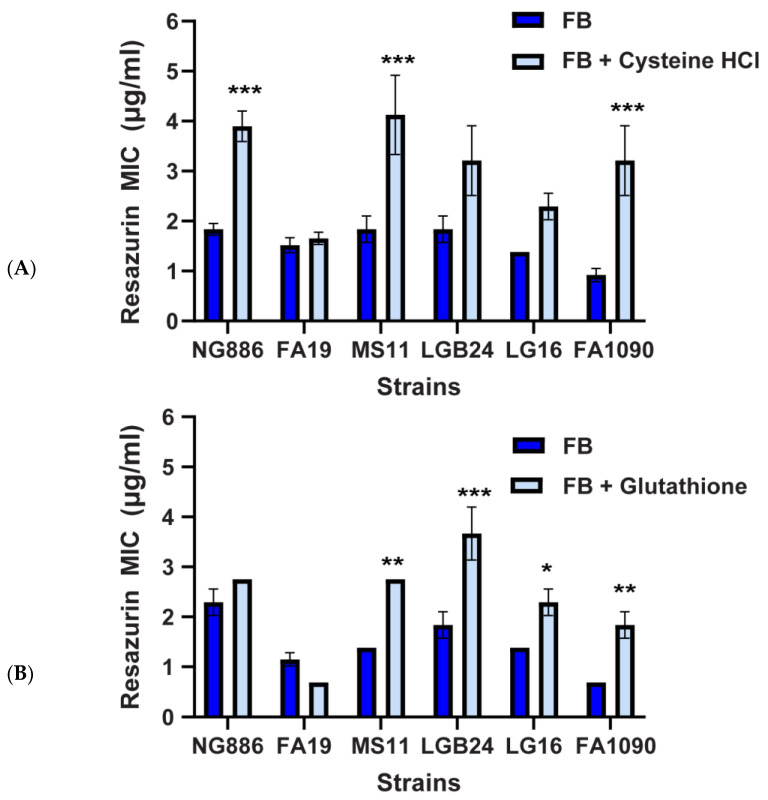
Oxidative stress contributes to *N. gonorrhoeae* susceptibility to resazurin. The MIC of resazurin for different *N. gonorrhoeae* clinical isolates is determined by a modified broth microdilution in the presence or absence of 3.2 mM cysteine hydrochloride (HCl) (**A**) or 2 mM reduced L-glutathione (**B**) under 20% oxygen. The MIC reported for each strain is the lowest concentration of each compound that prevents visible growth. Data shown are mean ± SEM from at least three independent experiments. No error bars are present if the SEM was equal to zero. Statistically significant differences in MIC were determined by two-way ANOVA followed by Tukey’s multiple comparisons test (*, *p* < 0.05; **, *p* < 0.01; ***, *p* < 0.001 comparing with or without cysteine HCl/glutathione for each strain).

Oxidative stress occurs when the accumulation of reactive oxygen species (ROS), such as hydrogen peroxide, superoxide, and hydroxyl radicals, overwhelm the cell’s detoxification mechanisms. ROS are produced endogenously, as by-products of aerobic metabolism, and exogenously from oxidants present in the environment [30]. High oxygen concentrations also induce ROS accumulation in bacteria: periplasmic superoxide levels rise proportionally to increases in dissolved oxygen concentrations [31]. Furthermore, as a redox-sensitive dye, resazurin has been shown to promote ROS generation [32,33]. Therefore, we hypothesized oxidative stress may contribute to the increased susceptibility of *N. gonorrhoeae* to resazurin at ~20% oxygen compared with 2% oxygen. To test this hypothesis, we determined the MIC of resazurin at ~20% oxygen in the presence and absence of two different antioxidants, cysteine hydrochloride (cysteine HCl) and reduced L-glutathione. These antioxidants were added at concentrations known to create reducing conditions (3.2 mM for cysteine HCl and 2 mM for L-glutathione [34,35]) and should scavenge and neutralize any ROS that are present, mimicking the lower oxidative stress environment at 2% oxygen. A two-fold increase in resazurin MIC was observed for three of the six *N. gonorrhoeae* strains tested (NG886, MS11, and FA1090) when cultivated in media supplemented with cysteine HCl compared wth untreated media (Figure 3). The MIC differences for NG886 in the presence and absence of cysteine HCl at ~20% oxygen (Figure 3) matched the MIC differences observed between 2% and ~20% oxygen (Figure 1). In the presence of a different antioxidant, reduced L-glutathione, four of the six *N. gonorrhoeae* strains had significantly higher resazurin MICs compared with cultures without glutathione (Figure 3). Two of these strains (MS11 and FA1090) also had elevated MICs in the presence of cysteine HCl while the reduced susceptibility of the other two strains (LGB24 and LG16) was specific to glutathione (Figure 3). This data suggest that oxidative stress plays a role in *N. gonorrhoeae* resazurin susceptibility at 2% and ~20% oxygen.

## 3. Discussion

*N. gonorrhoeae* has developed resistance to all antibiotics currently approved for gonorrhea treatment; with strains resistant to ceftriaxone, the last remaining monotherapy option, on the rise. The development of new antibiotics to combat this pathogen is essential and begins with the identification of compounds possessing robust bactericidal activity in vitro using standard antimicrobial susceptibility assays. However, several studies have shown that the clinical predictive value of these antimicrobial susceptibility assays is limited; largely due to their failure to consider the influence of host and environmental factors on the antimicrobial susceptibility of bacteria [36,37,38]. These factors include serum protein binding, inoculum size, antibiotic concentration, temperature, and interactions with the gut microbiota [39,40,41]. The data presented in this study identify oxygen concentration as another factor to consider when assessing the antimicrobial activity of a compound. Here, we showed that *N. gonorrhoeae* is more resistant to the antibiotic resazurin in low oxygen (2%) compared with atmospheric oxygen (~20%). While oxygen levels have been shown to influence the antibiotic susceptibility of select pathogens like *E. coli, P. aeruginosa*, and *S. aureus* [19,20,21], this is the first study, to our knowledge, that describes the effect of low oxygen on the antibiotic susceptibility of *N. gonorrhoeae*.

Resazurin exhibits robust bactericidal activity against *N. gonorrhoeae* in vitro, but has no therapeutic effect in a mouse model of gonorrhea [14]. Binding to serum albumin was previously identified as a factor contributing to resazurin’s lack of efficacy in vivo [14]. It is not the sole factor, however, as a resazurin analog that maintains its antimicrobial activity in the presence of BSA still failed to clear the gonococcal infection [14]. In this study, we show that the poor in vivo efficacy of resazurin also correlates with the reduced in vitro susceptibility of *N. gonorrhoeae* to resazurin at 2% oxygen, representing the hypoxic environment of the vagina [42]. Interestingly, *N. gonorrhoeae* is equally susceptible to other antibiotics, tetracycline and gentamicin, at low oxygen compared with atmospheric oxygen. This data suggest that the reduced susceptibility of *N. gonorrhoeae* to resazurin at 2% oxygen is not due to a broad resistance mechanism such as limiting uptake or drug efflux that affects multiple antibiotics, but a specific alteration in the bacterium or the compound, reducing resazurin’s efficacy. This hypothesis is further supported by data showing that various *N. gonorrhoeae* efflux pump mutants are also more resistant to resazurin at 2% oxygen compared with ~20% oxygen, similar to the clinical isolates tested. The MIC of resazurin for RD1 and BR54, which both lack a functional MtrCDE efflux pump, is greater than 4-fold higher in low oxygen conditions compared with atmospheric oxygen. However, there is a significant reduction in the resazurin MIC for BR54 at 2% oxygen compared with the parental FA19 strain. Given RD1 has a mutation in *mtrE* resulting in loss of expression of multiple efflux pumps (MtrCDE, FarAB-MtrE, and MacAB-MtrE) while BR54 has a mutation in *mtrD* resulting in loss of MtrCDE alone [43,44], this suggests efflux of resazurin through MtrCDE partially contributes to the enhanced resistance to resazurin at low oxygen. Furthermore, overexpression of the MtrCDE efflux pump in strain KH15 significantly increased the resazurin MIC in both oxygen conditions (~20% and 2%). Together, these data suggest resazurin is a substrate of the MtrCDE efflux pump, but the efflux of resazurin is not the main reason for the reduced susceptibility of *N. gonorrhoaea* to resazurin at low oxygen. Provided that MtrCDE confers resistance to a number of other hydrophobic drugs, dyes, and detergents, it is not surprising that it can also pump out resazurin [45]. Future studies will focus on understanding how resazurin interacts with this efflux pump and the role MtrCDE plays in the emergence of *N. gonorrhoeae* resistance to resazurin. 

Antibiotics exert their bactericidal effect by inhibiting key cellular processes such as cell wall synthesis, translation, and DNA replication [46]. Additionally, part of the lethality of these drugs comes from their ability to trigger ROS production [20]. While the mechanism by which resazurin kills *N. gonorrhoeae* has yet to be elucidated, it likely involves oxidative stress, as resazurin is known to promote ROS generation [32,33]. This hypothesis is supported by the data presented here showing *N. gonorrhoeae* is less susceptible to resazurin at decreasing concentrations of oxygen. Furthermore, the addition of antioxidants (reduced L-glutathione and cysteine hydrochloride) at 20% oxygen to scavenge and neutralize excess ROS resulted in increased resistance of *N. gonorrhoeae* to resazurin similar to cultivation in 2% oxygen. We are currently investigating ROS generation by *N. gonorrhoeae* in response to resazurin and the contribution of ROS-mediated cellular damage in the bactericidal activity of resazurin. 

Interestingly, an alkylated derivative of resazurin, resorufin pentyl ether (RPE), did not exhibit significant differences in antimicrobial activity under atmospheric and low oxygen conditions. RPE, like resazurin, is not effective at completely clearing *N. gonorrhoeae* infection in mice. This suggests other factors in addition to oxygen concentration and serum protein binding affect the antimicrobial activity of resazurin compounds [14]. Provided that resazurin and RPE are hydrophobic compounds, these drugs are likely being metabolized into more water soluble molecules that can be excreted. It is possible these metabolic modifications could reduce the antimicrobial activity of these compounds. From previous work and ongoing investigations, we know not all chemical modifications of resazurin yield active compounds [14]. The more characteristics we identify to be critical for the in vivo efficacy of resazurin compounds, the better we can design in vitro susceptibility assays to identify derivatives likely to have robust therapeutic efficacy in vivo.

## 4. Materials and Methods

### 4.1. Bacterial Strains and Reagents

*N. gonorrhoeae* strains used in this study are listed in Table 1 and were kindly provided by Drs. Ann Jerse (Uniformed Services University of the Health Sciences, Bethesda, MD, USA) and William Shafer (Emory University School of Medicine, Atlanta, GA, USA). Frozen stock cultures of bacteria were streaked onto chocolate II agar plates and incubated at 37 °C with 5% CO_2_ for 24–48 h. Resazurin sodium salt (Acros Organics, Morris Plains, NJ, USA), resorufin sodium salt (Sigma Aldrich, St. Louis, MO, USA), gentamicin sulfate (USBiological, Salem, MA, USA), tetracycline hydrochloride (Fisher Scientific, Waltham, MA, USA), reduced L-glutathione (Sigma Aldrich), and cysteine hydrochloride (Fisher Scientific) were dissolved in water, while resorufin pentyl ether (AnaSpec, Fremont, CA, USA) was dissolved in dimethyl sulfoxide (DMSO).

### 4.2. Antibiotic Susceptibility Testing

The minimum inhibitory concentrations (MICs) for resazurin derivatives and other antimicrobials against *N. gonorrhoeae* were determined by a modified broth microdilution assay, as described previously [14]. In brief, resazurin, resorufin, resorufin pentyl ether (RPE), tetracycline, and gentamicin were diluted in fastidious broth (FB) (Remel, Lenexa, KS) in 96-well microtiter plates (Corning Inc., Kennebunk, ME, USA) to yield concentrations ranging from 0 to 44 µg/mL for resazurin and resorufin, 24.8 µg/mL for RPE, and 32 µg/mL for tetracycline and gentamicin. In select experiments, FB was supplemented with either cysteine hydrochloride (3.2 mM) or reduced L-glutathione (2 mM). *N. gonorrhoeae* bacteria from chocolate II agar plates were suspended in tryptic soy broth (TSB) to a concentration of to 2 × 10^6^ CFU/mL and then 5 µL of this suspension (1 × 10^4^ CFU) was added to each well of the 96-well plate. Plates were incubated overnight at 37 °C in either a standard CO_2_ incubator (5% CO_2_, ~20% O_2_) or a tri-gas incubator (HeracellTM 150i, Thermo Scientific, 5% CO_2_, 2%O_2_, 93% N_2_). Bacteria from each well were then transferred to chocolate II agar plates using a 48-pin microplate replicator (Dan-Kar Corp., Woburn, MA, USA). Plates were incubated at 37 °C, 5% CO_2_ for 24–48 h. The MIC reported for each strain was the lowest concentration of each compound that prevented visible growth on chocolate agar.

### 4.3. Statistical Analyses

GraphPad Prism software 9.4.1 (GraphPad Software Inc., La Jolla, CA, USA) was used to determine the statistical significance of the data generated from this study. The statistical tests used and the *p* values obtained are presented in the individual figure legends.

## Figures and Tables

**Table 1:** Bacterial strains used in this study.

Strain	Description	Source or Reference
FA1090	Isolated from patient with disseminated gonococcal infection. Resistant to streptomycin.	Cohen et al. [47]
LGB-24	Isolated from urogenital tract. Resistant to tetracycline and penicillin; not a β-lactamase producer.	McKnew et al. [48]
NG886	Penicillin, tetracycline, and fluoroquinolone-resistant strain.	Cern et al. [49]
MS11	Isolated from a case of cervicitis. Overexpresses the MtrCDE multidrug efflux pump. Resistant to azithromycin and penicillin.	Swanson et al. [50]
FA19	Isolated from patient with disseminated gonococcal infection.	Mickelsen et al. [51]
LG-16	Isolated from urogenital tract. Resistant to penicillin, tetracycline, and azithromycin; β-lactamase producer.	Garvin et al. [52]
KH15	-T at MtrR binding site (*mtr-79*), overexpresses MtrCDE efflux pump	Hagman et al. [53]
RD1	FA19 *mtrE*::Km	Delahay et al. [43]
BR54	FA19 *mtrD-54*	Rouquette-Loughlin et al. [29]
CR28	FA19 *norM*::Km	Rouquette-Loughlin et al. [29]
CR29	BR54 *norM*::Km	Rouquette-Loughlin et al. [29]

## Data Availability

The original contributions presented in the study are included in the article, further inquiries can be directed to the corresponding author.
